# A novel lipoprotein lipase gene missense mutation in Chinese patients with severe hypertriglyceridemia and pancreatitis

**DOI:** 10.1186/1476-511X-13-52

**Published:** 2014-03-19

**Authors:** Tan-Zhou Chen, Sai-Li Xie, Rong Jin, Zhi-Ming Huang

**Affiliations:** 1 The Department of Gastroenterology and Hepatology, The First Affiliated Hospital of Wenzhou Medical University, Wenzhou, China; 2 Department of Epidemiology, The First Affiliated Hospital of Wenzhou Medical University, Wenzhou, China

**Keywords:** Lipoprotein lipase deficiency, LPL gene, Mutation, Hypertriglyceridemia, Pancreatitis

## Abstract

**Background:**

Alterations or mutations in the lipoprotein lipase (LPL) gene contribute to severe hypertriglyceridemia (HTG). This study reported on two patients in a Chinese family with LPL gene mutations and severe HTG and acute pancreatitis.

**Methods:**

Two patients with other five family members were included in this study for DNA-sequences of hyperlipidemia-related genes (such as LPL, APOC2, APOA5, LMF1, and GPIHBP1) and 43 healthy individuals and 70 HTG subjects were included for the screening of LPL gene mutations.

**Results:**

Both patients were found to have a compound heterozygote for a novel LPL gene mutation (L279V) and a known mutation (A98T). Furthermore, one HTG subject out of 70 was found to carry this novel LPL L279V mutation.

**Conclusions:**

The data from this study showed that compound heterozygote mutations of A98T and L279V inactivate lipoprotein lipase enzymatic activity and contribute to severe HTG and acute pancreatitis in two Chinese patients. Further study will investigate how these LPL gene mutations genetically inactivate the LPL enzyme.

## Background

Lipoprotein lipase (LPL) is a member of the lipase gene family, which includes lipases in the pancreas, liver, and endothelium and plays a critical role in metabolism of triglyceride (TG) by hydrolyzing TG-rich lipoprotein to free fatty acids using apolipoprotein C-II (apoC-II) as a cofactor. Alterations or mutations in the LPL gene could result in massive accumulation of chylomicrons and profound fasting hypertriglyceridemia (HTG) [1]. Patients with severe HTG (TG > 11.3 mmol/l) usually suggest genetic reasons and show clinical features of recurrent attacks of pancreatitis, hepatosplenomegaly, lipemia retinalis and eruptive xanthomas. The primary severe HTG may be caused by gene mutations, including deficiency of LPL, apoC-II and apoA-V, correspondingly to mutations of LPL, APOC2, and APOA5 genes, respectively [2,3]. Recently, other two novel proteins were identified to be essential for proper LPL function, i.e., lipase maturation factor1 (LMF1) and glycosylphosphatidylinositol-anchored high-density lipoprotein-binding protein 1 (GPIHBP1); thus, they are of importance for studying HTG [4,5], although mutations of LPL gene are responsible for the most common form of primary severe HTG.

Human LPL gene is composed of 10 exons and spans approximately 30 kb on chromosome 8p22 encoding a protein with 448 amino acids [6]. LPL gene mutations include insertion, duplication, deletion, nonsense, frameshift, or missense mutations. Most LPL gene mutations-caused LPL deficiency are due to missense mutations, although other types of mutations have been previously reported [7]. To date, there are over a hundred LPL gene mutations reported in the literature to cause clinical hyperchylomicronemia syndrome in the Human Gene Mutation Database. In this study, we described a novel LPL gene missense mutation (L279V) in two Chinese patients with severe HTG and acute pancreatitis.

## Subjects and methods

### Subjects

In the present study, a 28-year-old female Han Chinese with her first pregnancy during the third trimester was admitted to our emergency room and diagnosed with acute pancreatitis. Several days prior to this episode, lactescent levels were elevated in her serum however this was not noted. When admitted, blood tests revealed extremely high serum levels of HTG (TG 79 mmol/l, while normal ranges are 0.0-1.7 mmol/l). Following immediate cesarean section, she gave birth to a healthy baby girl and then was treated with dietary restriction and supportive treatment for 28 days, then discharged from the hospital.

Meanwhile, the 58-year-old mother was also admitted to our hospital with a diagnosis of acute pancreatitis. Including this hospitalization, she had two previous episodes of abdominal pain and was diagnosed with acute pancreatitis after intake of fatty food. The plasma levels of total triglyceride were 25.4 and 16.3 mmol/l, respectively at these episodes. The proband’ mother was married to her father at age 22 through a consanguineous marriage. Because these two patients presented with severe HTG and acute pancreatitis, they were strongly suggestive of an inherited condition.

Thus, we included not all but five of their family members, i.e., the father, two aunts, one uncle and one sister in our study. They all were shown to have mild hyperlipidemia but no history of diabetes mellitus, abdominal pain or pancreatitis. In addition, we also recruited an additional 43 healthy individuals and 70 unrelated HTG subjects as controls for this study.

This study was approved by the Ethics Committee of Wenzhou Medical University and signed informed consent was obtained from all subjects participated in this study.

### Measurement of plasma lipase and LPL mass and activity

Blood samples were collected into Na-EDTA tubes 15 min following an intravenous heparin (60 IU/kg body weight) injection in these patients and in 10 healthy controls, including 5 men and 5 women after an overnight fast. LPL enzyme activity in post-heparin treatment was measured as described previously [8]. LPL mass was measured using a sandwich-ELISA with a Human Lipoprotein Lipase ELISA Kit (Nanjing Jiancheng, Nanjing, China).

Plasma lipid concentrations were also measured using standardized enzymatic methods as described previously [9]. The serum was diluted with saline solution (1:5) before measurement of HDL-C due to the high TG concentration for the daughter.

### PCR-amplification and DNA-Sequencing of candidate genes

Genomic DNA was extracted from 2 ml whole blood samples using a Gentra Puregene Blood kit according to the manufacturers’ instructions (Qiagen, Dusseldorf, Germany). All coding regions and the intron-exon boundaries of LPL (NM_000237), APOC2 (NM_000483), APOA5 (NM_052968), LMF1 (NM_022773), and GPIHBP1 (NM_178172) genes were PCR-amplified and bidirectionally DNA-sequenced using the Sanger method based on dideoxy chain-termination technology, as described previously [10,11].

### Screening for LPL gene mutations

The screening of the novel LPL variant L279V and the known variant A98T were performed in 43 healthy individuals and 70 HTG subjects using an i-plexGOLD genotyping assay of the MassARRAY mass-spectrometry system, following the protocol recommended by the manufacturer (Sequenom, Santiago, USA).

### Species examination

We examined evolutionary conservation of the L279 amino acid residue across various species from chimpanzee (a close evolutionary relative) to wild goose and zebra fish (both distant evolutionary counterparts).

## Results

### Genetic and biochemical features of the patients

**Figure 1:**
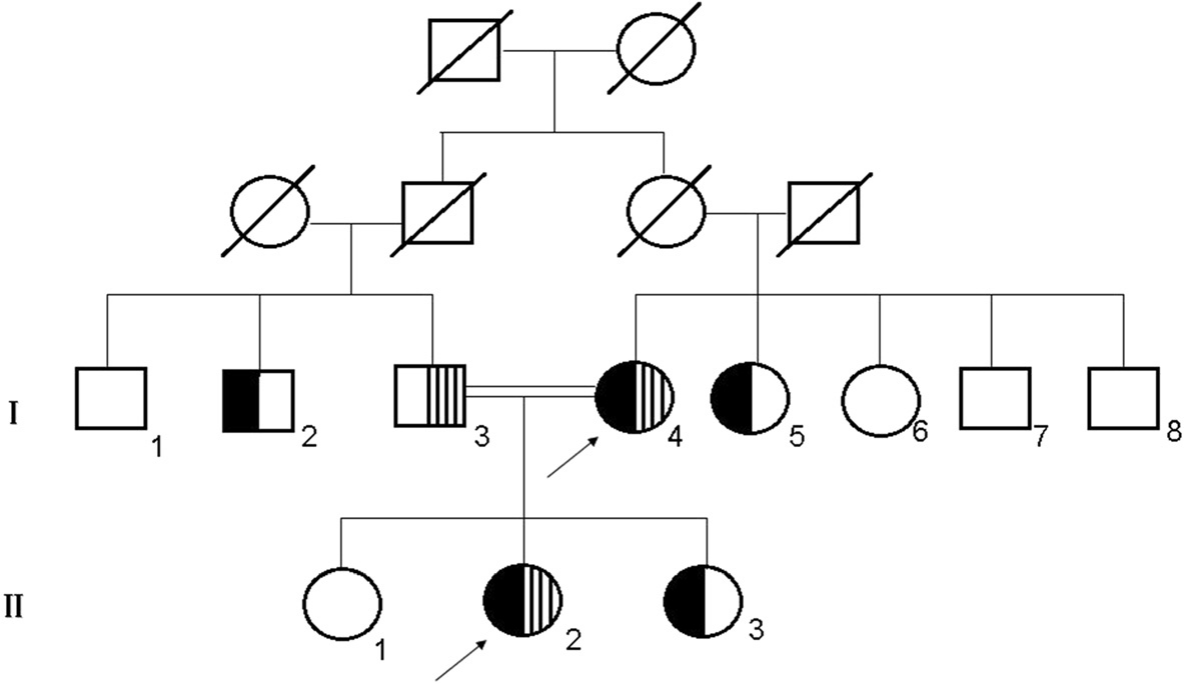
**Pedigree of the patient’s family.** Black symbols represent the presence of a A98T mutation. Shaded symbols represent the presence of a L279V mutation. The arrow indicates probands. Parental consanguinity is indicated by a double line. I 1, I 7, I 8 and II1 were not a part of the study.

**Figure 2:**
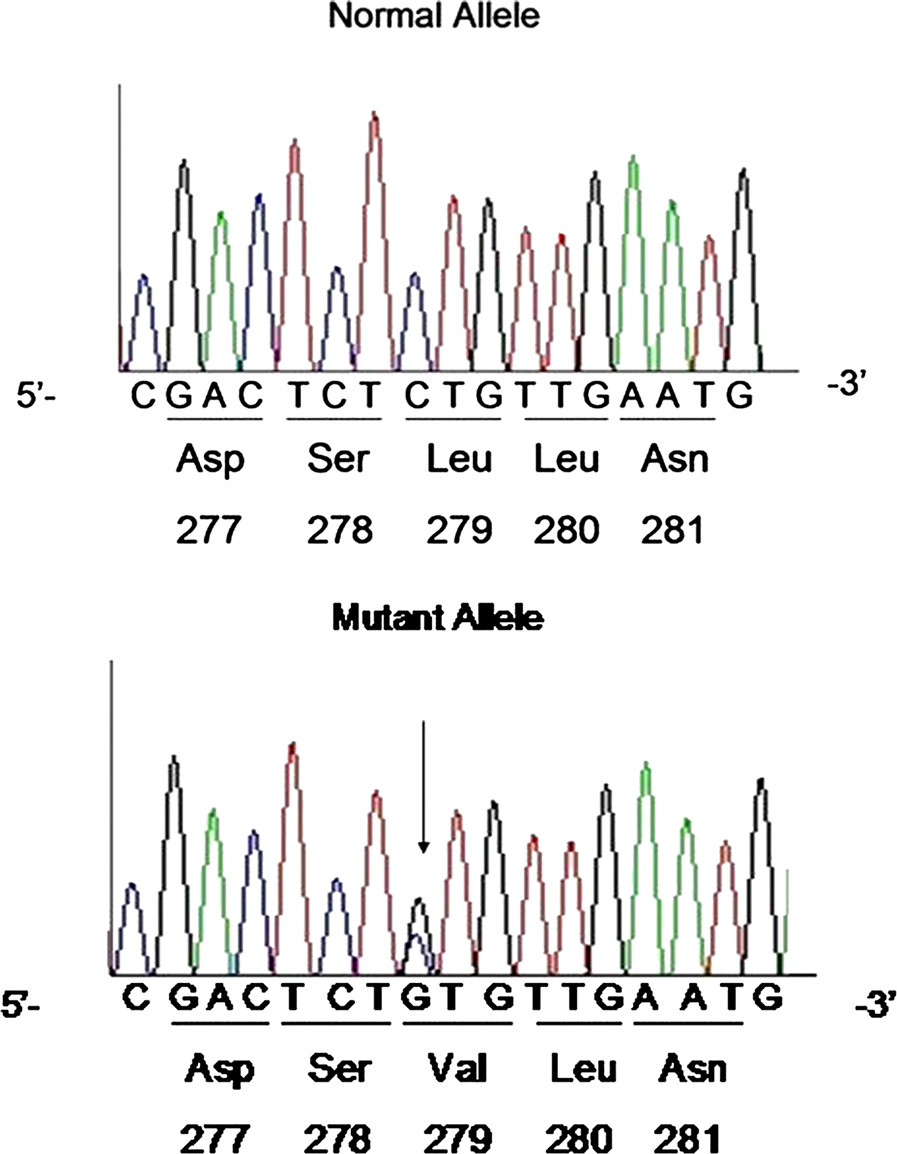
DNA sequence of LPL gene exon 6 mutations.

**Figure 3:**
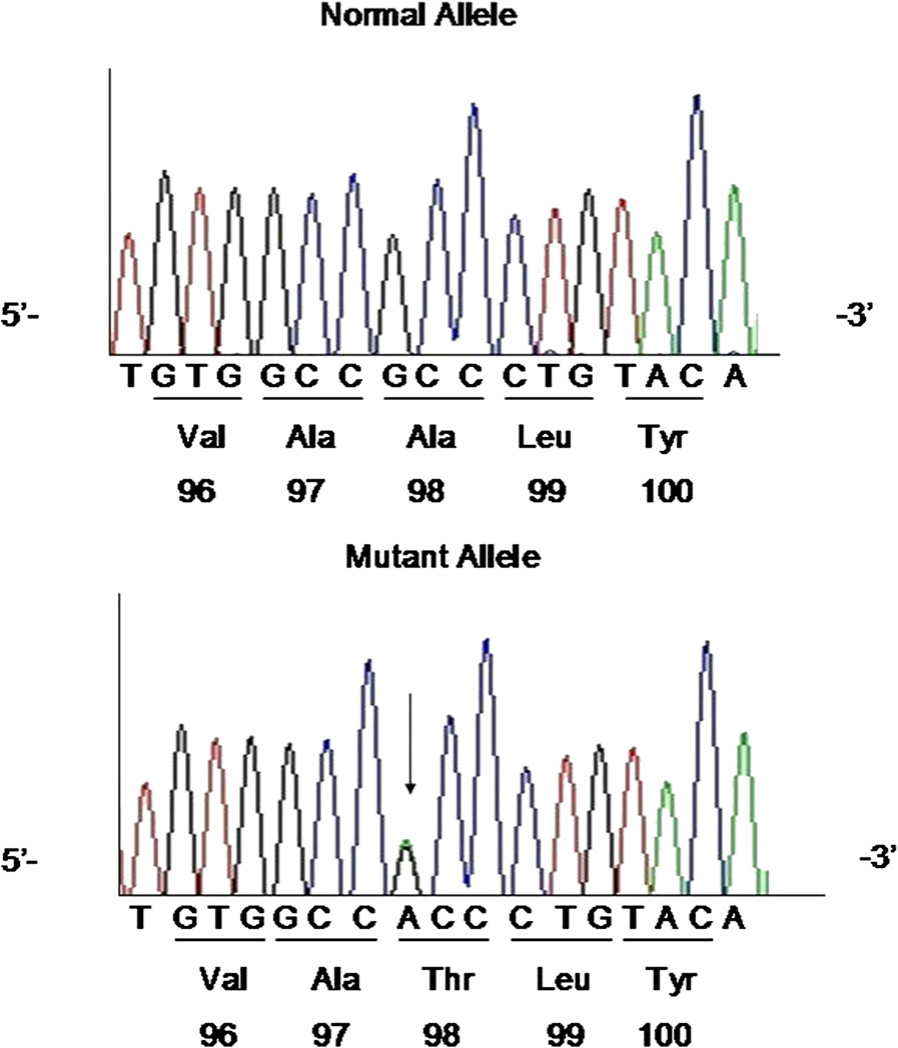
DNA sequence of LPL gene exon 3 mutations.

Pedigree of the probands is shown in Figure 1. Mutational analyses of the LPL, APOC2, APOA5, LMF1, and GPIHBP1 genes showed that both patients had the same compound heterozygote of the LPL gene for a novel missense mutation (L279V) and a known missense mutation (A98T). One unrelated HTG patient was also found to be heterozygote of the p.(L279V) variant. The novel missense mutation L279V (p.L279V, rs371282890) at exon 6 is a CTG → GTG change in codon 279 of the LPL gene, leading to L → V amino acid substitution in the LPL protein (Figure 2).The known missense mutation of LPL A98T (p.A98T, rs145657341) is a GCC → ACC change in codon 98 of the LPL gene, leading to an A → T amino acid substitution (Figure 3). The father (I3) was found to be a carrier of L279V, while the uncle (I2), one aunt (I5) and the elder sister (II3) were carriers of A98T. I2, I5, I6 also have S447X mutations. There were no mutations found in APOC2, APOA5, GPIHBP1 or LMF1.

**Figure 4:**
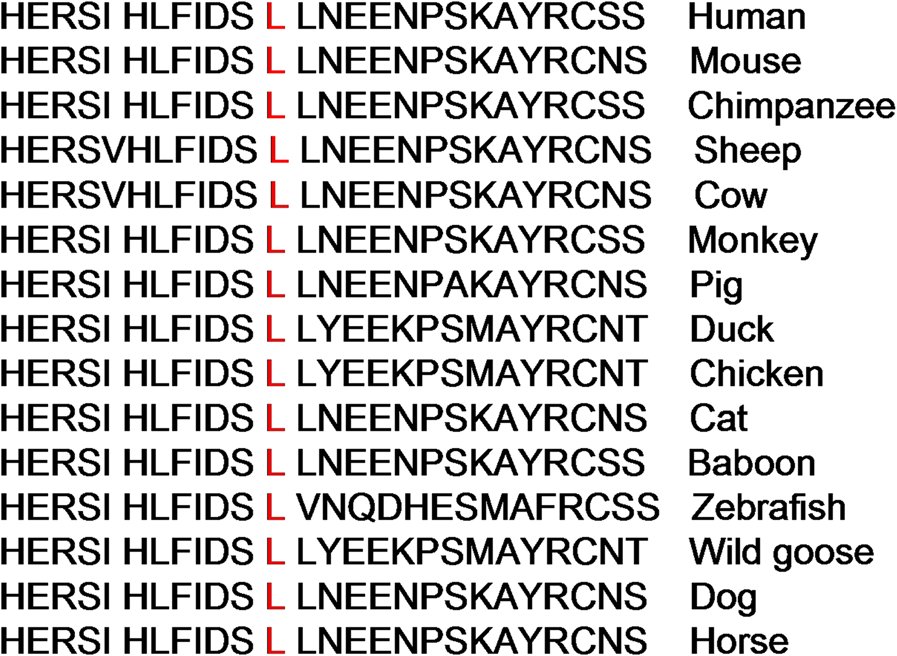
Comparison of evolutionary conservation of the L279 amino acid residue in various species.

LPL mutations and biochemical features of these two patients and their family members are shown in Table 1. However, in cross species comparison, we found that L279 residue is very conserved across the species (Figure 4).

**Table 1:** LPL mutations and biochemical features of the 2 probands and their family members

**Subject**	**Status**	**Genotype**	**Sex/age**	**BMI (kg/m**^**2**^**)**	**Plasma lipid levels (mmolL**^**-1**^**)**			
					**TC**	**TG**	**HDL-C**	**LDL-C**
I2	Uncle	A98T/S447X	M/69	24.2	5.36	4.53	1.02	1.78
I3	Father	L279V	M/63	23.5	5.13	2.03	1.21	1.53
I4	Mother(proband1)	L279V/A98T	F/58	19.2	7.99	16.33	0.54	1.28
I5	Niece	A98T/S447X	F/69	21.6	5.07	5.77	1.12	1.67
I6	Niece	S447X	F/66	20.1	5.72	5.45	1.36	2.06
II2	Daughter(proband2)	L279V/A98T	F/28	19.6	38.4	79.0	0.61	2.48
II3	Daughter	A98T	F/32	19.8	5.32	5.13	1.25	1.82

BMI, body mass index; TC, total cholesterol; TG, total triglyceride; HDL-C, high-density lipoprotein cholesterol; LDL-C, low-density lipoprotein cholesterol.

### Screening for LPL gene mutations in HTG subjects

Among the 70 unrelated HTG subjects, we found one HTG subject who carried the L279V mutation. This patient had an episode of acute pancreatitis and the TG levels were 15.3 mmol/l. Thus, we further analyzed and DNA-sequenced his genomic DNA for LPL, APOC2, APOA5, LMF1 and GPIHBP1 genes. The data showed that a missense mutation of GPIHBP1 gene (p.C14F, rs11538389) with heterozygosity of the gene. However, both A98T and L279V mutations were not found in any healthy individuals and other HTG subjects.

### Detection of post-heparin LPL activity and mass levels in patients and controls

We then detected post-heparin LPL activity and mass levels in patients and controls and found that compared to normal controls, the post-heparin LPL activity of the proband and her mother were reduced by 55.4% and 32.1%, respectively, although their plasma LPL mass was close to the normal controls (data not shown).

## Discussion

In the current study, we showed two-related patients who manifested massive HTG and acute pancreatitis with a low LPL post-heparin activity. Both probands present the same LPL genotype with a compound heterozygote for a known missense mutation A98T in exon3 and a novel missense mutation L279V in exon 6 of LPL gene. Moreover, the novel mutation L279V was also found in one of 70 HTG individuals.

To date, the majority disease-causing LPL gene mutations occurs at exon 4, 5 and 6 residues (117–312), which constitute a large N-terminal domain (residues 1–312) of the enzyme and this region is the most conserved and important for LPL catalytic functions [12]. Exon 6 (residues 232–313) encodes two structurally relevant disulfide bridges (Cys278-Cys283 and Cys264-Cys275) for the binding of heparin [13]. The novel mutation L279V constitutes one of these two disulfide bridges (Cys278-Cys283), which is important for catalytic function of heparin binding. Both patients had low post-heparin LPL activity but normal levels of mass, further demonstrating this possibility.

Furthermore, we also assessed the L279V mutation disease-causing potential by comparing it across various species. We found that the L279 site is indeed conserved throughout evolution from chimpanzee to zebra fish, suggesting that this residue may play a critical role in LPL function. In the current study, we found a total of four carriers of L279V mutation including the two patients and one family member, and an unrelated HTG individual, all of whom were heterozygotes for this missense mutation. This novel LPL mutation (L279V) was submitted to NCBI and the Submitter SNP accession number is ss#550039488. In addition, the known LPL gene mutation A98T has been described previously with lipoprotein lipase deficiency in the clinic [14]. To date, there were three family members found to carry this mutation, one with a single mutation and others with compound heterozygous for another nonsense mutation S447X of LPL.

Patients with two defective LPL alleles will have no or markedly reduced LPL activity, thus, homozygous or compounds of heterozygous mutations lead to severe HTG while one defective LPL allele may have normal to moderately increased blood levels of fasting triglyceride [15-17]. In this study, the mother and daughter had the same LPL genotype with compound heterozygous mutations of A98T and L279V; thus, both manifested massive HTG and acute pancreatitis. In contrast, the family members who carried a single mutation either A98T or L279V, only presented mild HTG and had no history of acute pancreatitis. Moreover, another unrelated HTG individual, who had the compound heterozygote L279V and a known GPIHBP1 mutation, suffered from a severe HTG and acute pancreatitis.

In the current study, we found that two family members had compound LPL gene A98T and S447X mutations, but they also had mild elevated blood TG levels. The reason for this is unclear, but may be because of the special mutation S447X. In a previous study, LPL S447X mutation was shown to have a naturally occurring gain-function [18]. This LPL mutation S447X is the only mutation that has a protective effect against the development of HTG and coronary heart disease by decreasing plasma TG levels in the clinic and increasing HDL production [19]. Thus, gene therapy using the S447X variant has shown therapeutic promise in the study of LPL deficiency [20].

## Competing interests

There are no potential competing interests to declare.

## Authors’ contribution

Z-MH planned the study protocol, took care about the patients and coordinated the research, T-ZC took part in the data analysis and drafted the manuscript acquisition of data, S-LX conceived of the study, participated in its coordination, revised it critically and prepared the final version of the manuscript, RJ participated in the study coordination and took part in the data analysis. All authors read and approved the final manuscript.
